# Post-Transplant Cyclophosphamide-Based GVHD Prophylaxis After Peripheral Blood Stem Cell HLA Identical Transplantation in Patients with Lymphoma: A Prospective Observational Study

**DOI:** 10.3390/life15030393

**Published:** 2025-03-03

**Authors:** Stefania Bramanti, Daniela Taurino, Filippo Magri, Chiara De Philippis, Barbara Sarina, Luca Castagna, Laura Giordano, Jacopo Mariotti, Daniele Mannina, Armando Santoro

**Affiliations:** 1 Bone Marrow Unit, IRCCS Humanitas Research Hospital, Humanitas Cancer Center, Rozzano, 20089 Milan, Italy; stefania.bramanti@humanitas.it (S.B.); chiara.de_philippis@humanitas.it (C.D.P.); barbara.sarina@humanitas.it (B.S.); jacopo.mariotti@humanitas.it (J.M.); daniele.mannina@humanitas.it (D.M.); armando.santoro@humanitas.it (A.S.); 2 Immunohematology and Transfusion Medicine Unit, IRCCS San Raffaele Scientific Institute, 20089 Milan, Italy; magri.filippo@hsr.it; 3 Bone Marrow Unit, Ospedale Villa Sofia Cervello, 90146 Palermo, Italy; l.castagna@villasofia.it; 4 Department of Biomedical Sciences, Humanitas University, Pieve Emanuele, 20072 Milan, Italy; laura.giordano@humanitas.it

**Keywords:** allo-SCT, GVHD, prophylaxis, cyclophosphamide, lymphoma

## Abstract

Allogeneic stem cell transplantation (allo-SCT) from HLA-identical donors (HLAid) could be an effective salvage treatment for relapsed/refractory lymphoma. In this setting, standard graft-versus-host disease (GVHD) prophylaxis is based on cyclosporine and methotrexate, with the addition of anti-thymocyte globulin, at least for matched, unrelated donors. Promising data using post-transplant cyclophosphamide (PT-Cy) have been reported from retrospective studies in patients receiving allo-SCT from HLAid donors. Here, we report the results of a single-center, prospective observational study exploring the main outcomes of GVHD prophylaxis based on PT-Cy in 27 patients receiving HLAid donor transplantation for relapsed/refractory lymphoma. With a median follow-up of 38 months, 3-year GVHD-relapse-free survival and PFS and OS were 70.4%, 81.5%, and 88.9%, respectively. The 1-year cumulative incidence (CI) of non-relapse mortality (NRM) was 7.4%. The 6-month CI of acute GVHD was 7.4%, and the 1-year CI of extensive chronic GVHD was 7.7%, with no grade IV GVHD events or deaths from GVHD. Relapse was reported in three patients (1-year relapse incidence: 11%), and two died of progressive disease. No graft failure was observed. This study shows that PT-Cy may be an effective strategy to prevent GVHD in patients with lymphoma receiving HLAid transplantation. It is associated with low NRM and reasonable disease control.

## 1. Introduction

Before the introduction of CAR-T therapy, allogeneic stem cell transplantation (allo-SCT) was the only strategy capable of achieving long-term survival in patients with relapsed or refractory lymphoma after more than two lines of chemotherapy and/or autologous transplantation and poor prognosis. HLA identical donor (HLAid), sibling or unrelated (UD) peripheral blood stem cells (PBSCs), is considered the most suitable donor source [1]. With the introduction of CAR-T-cell therapy for treating relapsed/refractory lymphoma, the clinical course of these patients has undergone further changes. The role of allo-SCT in patients relapsed or refractory to CAR-T-cell therapy remains controversial. However, it may represent these patients’ only potential curative option, offering a promising therapeutic avenue in the context of otherwise limited treatment options [2,3]. The only recently published prospective trial on allo-HCT for relapsed/refractory aggressive non-Hodgkin lymphoma is DSHNHL 2004-R3 (NCT00785330) [4], which enrolled 84 patients. With 55% of the patients being chemorefractory at allo-HCT, PFS and OS were 45% and 52% at 1 year and 39% and 42% at 3 years post-transplant. One-year relapse/progression incidence was 29%, with no relapse event occurring thereafter.

In this setting, prophylaxis for graft-versus-host disease (GVHD) was historically based on cyclosporine (CSA) and methotrexate (MTX), with anti-thymocyte globulin (ATG) in UD donors. With this approach, the expected cumulative incidence (CI) of acute GVHD (aGVHD) grade II-IV was 35%, and extensive chronic GVHD (cGVHD) was 25% [5]. In the haploidentical donor transplant setting, T-cell replete grafts with post-transplant cyclophosphamide (PT-Cy) have become the standard GVHD prophylaxis for patients with hematological malignancies [6,7,8]. Several studies have evaluated the use of PT-Cy as GVHD prophylaxis in patients who underwent HLA-matched transplantation in leukemia patients [9,10,11,12,13,14,15,16,17,18,19]. Collectively, these studies showed a lower incidence of acute and chronic GVHD compared with standard calcineurin inhibitors (CNI)-based GVHD prophylaxis with or without ATG in the HLA-matched–related or –unrelated allo-SCT [13,14]. However, information on the application of this approach in patients with lymphoid disease is scant, and more data are needed to assess the role of PT-Cy as GVHD prophylaxis in this patient population.

Here, we present a single-center prospective observational study to evaluate GVHD-relapse-free survival (GRFS) using a GVHD prophylaxis based on the combination of PT-Cy, CSA, and mycophenolic acid (MPA) according to the Baltimora-modified platform [8] in patients diagnosed with relapsed/refractory (R/R) lymphoma who underwent allo-SCT from HLAid donors, both familiar and unrelated, after a reduced-intensity (RIC)/non-myeloablative (NMA) conditioning regimen.

## 2. Materials and Methods

### 2.1. Setting and Design

This single-arm, double-stage, open-label, prospective observational study was conducted from March 2018 to November 2021 and evaluated the patients diagnosed with R/R lymphoma receiving PBSC HLAid transplantation conditioning with RIC/NMA regimen and GVHD prophylaxis with PT-Cy, CSA, and MPA.

The institutional review board of the IRCCS Humanitas Research Hospital approved the study. Patients provided informed consent for the collection of their clinical data. All procedures were performed by the Ethical Standards of the Responsible Committee on Human Experimentation (institutional and national) and with the Helsinki Declaration of 1975, as revised in 2008.

### 2.2. Patients

All patients had a histologically confirmed lymphoma diagnosis in complete remission (CR) or partial remission (PR) after the last chemotherapy line. Therefore, the indications for allo-SCT were primary refractory lymphomas, relapse after autologous transplantation, or PBSC mobilization failure.

Other inclusion criteria were as follows: 18–70 years of age; heart ventricular ejection fraction > 40%; DLCO and FEV1 > 50% predicted; total bilirubin ≤ 2.5 mg/dL; ALT, AST, and alkaline phosphatase < 5 × upper limit of normal (ULN); creatinine clearance or GFR by Cockroft–Gault formula > 50 mL/min/1.73 m^2^; Karnofsky performance score ≤ 60%.

Exclusion criteria were haploidentical or mismatched unrelated donor (MMUD) transplantations and any medical condition contraindicating allo-SCT.

### 2.3. Endpoints

The study’s primary endpoint was the proportion of patients without GRFS at one year. The secondary endpoints were incidence of engraftment, chimerism, aGVHD grade II-IV and III-IV, cGVHD, NRM, relapse, progression-free survival (PFS: defined as the time from enrollment to progression of disease, death, or loss to follow-up; whatever occurred first), overall survival (OS: defined as the time from enrollment to death or loss to follow-up; whatever occurred first), and incidence of infection.

### 2.4. Conditioning Regimen and GVHD Prophylaxis

The choice of conditioning regimens followed local guidelines and took into consideration the patient’s age, the hematopoietic cell transplantation-comorbidity index [20], disease risk index (DRI) [21], and disease status at transplant. Three main conditioning regimens were used: (a) NMA regimen (Cy 14.5 mg/kg on days −6 and −5, fludarabine 30 mg/m^2^ from day −6 to day −2, and low-dose total body irradiation (TBI) (2 Gy) on day −1); (b) RIC regimen (either thiotepa 5 mg/kg on day −7, busulfan 65 mg/m^2^ on day −6 and day −3, and fludarabine 40 mg/mq from day −6 to day −3; since 2019 the patients received busulfan target AUC = 16.000 μmolar × minutes [22,23,24]); and (c) RIC regimen (thiotepa 6 mg/kg BID on day −5, cyclophosphamide 30 mg/kg from day −4 to day −3, and fludarabine 30 mg/m^2^ from day −4 to day −3).

The GVHD prophylaxis regimen consisted of PT-Cy 50 mg/kg intravenous (IV) administered on days +3 and +4, CSA 3 mg/kg from day +5 to day +100, and MPA 45 mg/kg divided into three doses administered orally from day +5 to day +35. CSA dose was progressively reduced from day +100 to day +180.

Subcutaneous granulocyte colony-stimulating factor (G-CSF) was used from day +5 at 5 μg/kg/day. Prophylaxis against bacterial, viral, and fungal agents was administered according to local guidelines [25]. In addition, monitoring for CMV and EBV reactivation by PCR was performed twice a week during the early period (+15 to day +100) and then weekly until day +180. Letermovir was approved in Italy in 2018. However, according to the AIFA restrictions, only 26% of the patients in the study performed prophylaxis with letermovir based on donor/recipient CMV status.

### 2.5. Stem Cell Source and Donors

Family members who were potential donors were HLA typed at the HLA-A, HLA-B, HLA-DRB1, and HLA-DQB1 loci at a high-resolution level. In addition, selected donors were typed at the HLA-C locus at a high-resolution level. The selection of donors in case of multiple-choice availability was based on weight, age, blood group, and CMV serology matches.

Donors underwent BM harvest under general anesthesia or were mobilized by subcutaneous G-CSF at 10 mg/kg/day for 5–6 days. In addition, unmanipulated bone marrow (BM) and PBSC were infused on day 0.

### 2.6. Engraftment Definition, GVHD, and Treatment Response Evaluation

White blood count recovery was defined as the first three consecutive days with an absolute neutrophil count of 0.5 × 10^9^/L since G-CSF was interrupted. Platelet recovery was defined as a count of 20 × 10^9^/L, with no transfusions required during the preceding seven days. Graft failure is defined as the lack of hematopoietic cell engraftment following allo-SCT. The MAGIC criteria [26] were used to grade aGVHD, and the NIH criteria [27] were used to grade cGVHD. Disease assessment was performed with a PET scan before the transplant for all patients [28,29]. In addition, pre-transplant CR patients were evaluated with a CT scan at 100 days’ post-transplant and subsequently every three months up to 1 year. Those in PR with PET scan at 100 days’ post-transplant and then with CT scan at the same time points. Chimerism was evaluated on peripheral blood 30 days after transplantation. In cases of male/female gender mismatch, the chimerism is evaluated by karyotype analysis and, in the absence of mismatch, by studying short tandem repeats by multiplex-PCR [30].

### 2.7. Statistical Analysis

The data were collected within a prospective clinical trial in which the sample size was not reached. Here, we present analyses of the primary outcome for exploratory purposes. Data were summarized as frequencies and proportions for categorical variables and median and range for continuous variables. Kaplan–Meier method was used to estimate survival and to depict corresponding curves. CI was used to estimate aGVHD, cGVHD, and NRM. In the data analysis, given the small sample size and number of events, the restricted mean survival time (RMST) was calculated for GRFS, with bootstrap resampling performed for estimation. All analyses have been performed using SAS version 9.4 (SAS Institute Inc., Cary, NC, USA).

## 3. Results

### 3.1. Patients, Transplant, and Donor Characteristics

From March 2018 to 27 November 2021 patients were enrolled; the main patients, donors, and transplant characteristics are shown in Table 1.

One-third of patients (*n* = 9; 33%) were affected by Hodgkin lymphoma (HL), and two-thirds (18; 67%) by non-Hodgkin lymphoma (NHL).

Most patients were candidates for allo-SCT because they were primary refractory (*n* = 12, 45%) or relapsed after autologous transplantation (*n* = 10, 37%). Patients underwent a median of four lines of prior chemotherapy, and only one patient underwent an allogeneic transplant as consolidation following first-line therapy for T-prolymphocytic leukemia, achieving complete remission after alemtuzumab treatment. All patients with HL were previously exposed to checkpoint inhibitors and brentuximab vedotin. Among patients with DLBCL, only one underwent transplantation in relapse post-CAR T-cell therapy, while three did so after treatment with glofitamab. The majority of patients with mantle cell lymphoma were chemotherapy-refractory and achieved complete response after ibrutinib.

Most patients were in CR (*n* = 22, 81%). PBSC was the transplantation source for all but one patient, and the conditioning regimen was TBF for 78% of the patients (*n* = 21); 67% (*n* = 18) of HLA-matched donors were siblings.

### 3.2. GVHD-Relapse-Free Survival, PFS, OS

**Figure 1:**
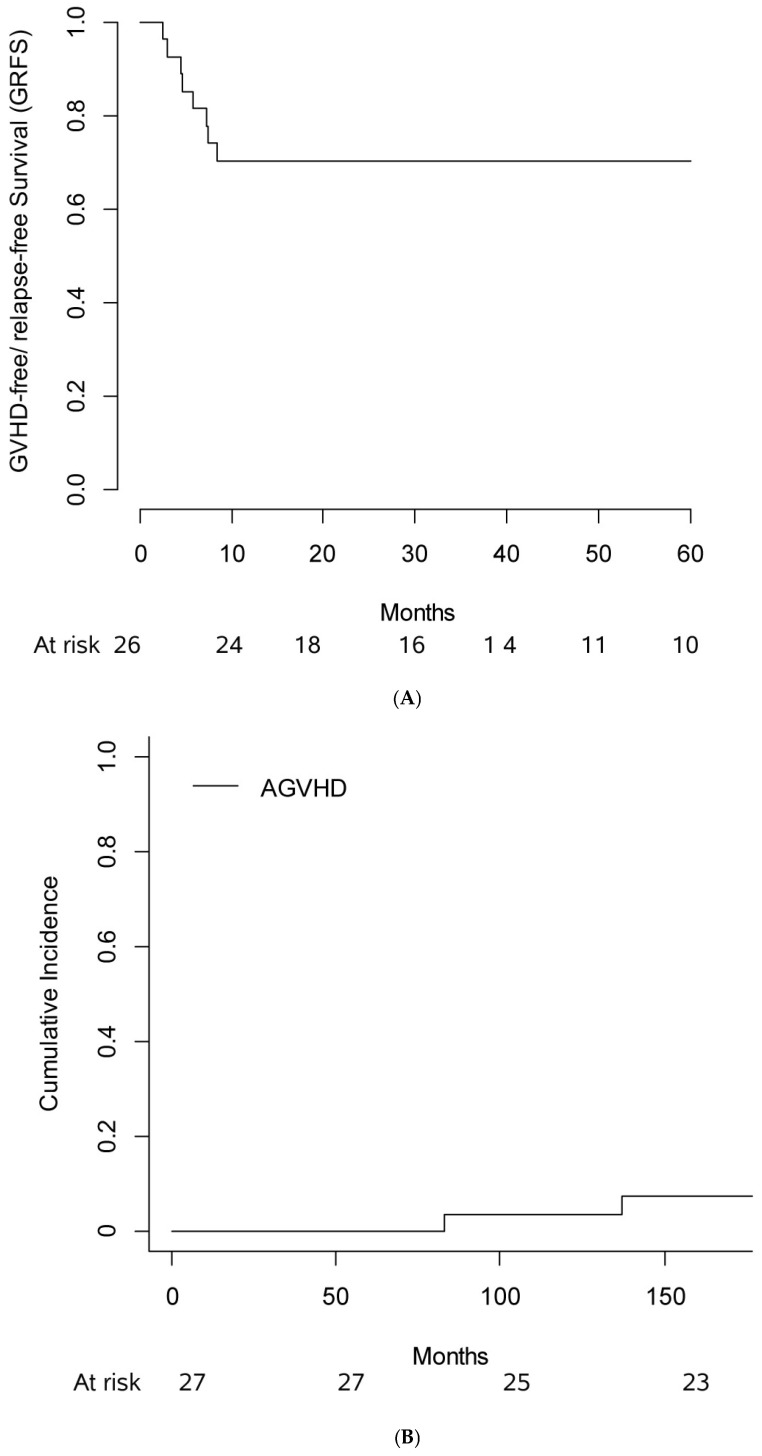

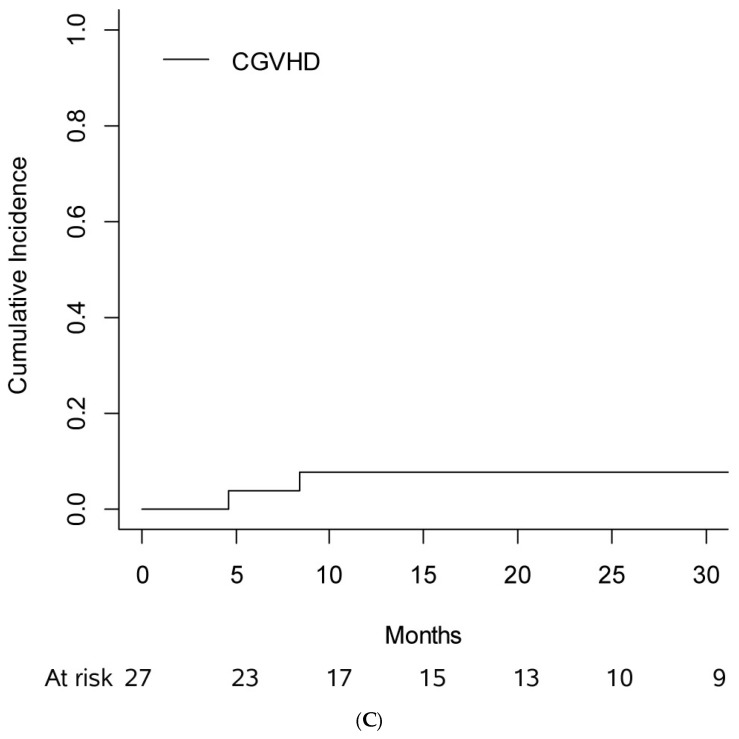
(**A**) 6-month CI of aGVHD grade II-IV, (**B**) 1-year CI of extensive cGVHD, and (**C**) 1-year GRFS.

With a median follow-up of 37 months (IQ range: 25; 52), the 3-year PFS was 81.5% (CI 95%: 61.1; 91.8%), the 3-year GRFS was 70.4% (CI 95%: 49.4; 84%, Figure 1A), and the 3-year OS was 88.9% (CI 95%: 69.4; 96.2%). GRFS, RMST at 2 years was 18.5 months (CI 95%: 14.9; 21.4%).

### 3.3. Hematopoietic Recovery

No graft failures were observed. At 100 days, all but one patient had complete chimerism. The patient with mixed chimerism achieved complete chimerism at subsequent follow-up after cyclosporine discontinuation.

The median time for neutrophil and platelet recovery was 21 days (IQ range: 19; 21) and 23 days (IQ range: 17; 28), respectively.

### 3.4. GVHD

The 6-month CI of aGVHD grade II-IV was 7.4% (CI 95%: 0; 17.5%) (Figure 1B). In particular, one patient had grade II aGVHD (skin stage III, upper gastrointestinal stage I) and one grade III aGVHD (liver stage III); no grade IV aGVHD was observed. The 1-year CI of severe cGVHD was 7.7% (CI 95%: 0, 18.1%) (Figure 1C), with two patients experiencing moderate cGVHD. No patient died of GVHD.

### 3.5. NRM, RELAPSE

The 1-year CI of NRM was 7.4% (CI 95%: 0; 17.5%); two patients died of encephalitis, one from Escherichia coli and the other from an unknown cause. Only two patients experienced disease relapse at 77- and 220 days post-transplant, respectively. Overall, the 1-year cumulative incidence of relapse was 11.1% (CI 95%: 0; 23.2%), with two patients dying of progressive disease and the other presently in CR following CAR-T-cell therapy.

### 3.6. Infections

About 89% (CI 95%: 71; 97.6%, *n* = 24) of patients experienced an infectious event during the follow-up period. The most frequent symptoms were febrile neutropenia, pneumonia, CMV reactivations, BK cystitis, and sepsis. In more detail, ten patients (37%, CI 95%: 19; 58%) reported a bacterial infection, seven (26%, CI 95%: 11; 46%) had a viral infection, and four (15%, CI 95%: 0; 34%) showed a fungal infection.

Only one patient died of an infective complication (*E. coli* encephalitis at 160 days’ post-transplantation).

CMV reactivation was documented in seven patients (26%, CI 95%: 11; 46%) at a median time of 32 days’ post-transplantation. Only two patients developed a CMV infection, specifically gastritis and pneumonia, 36 and 35 days after the transplant, respectively. Furthermore, three patients (11%, CI 95%: 0; 29%) developed HHV-6 gastritis at a median time of 58 days’ post-transplant. BK virus cystitis was documented in three patients (11%, CI 95%: 0; 29%) at a median time of 41 days’ post-transplantation, with no grade ≥ III.

## 4. Discussion

Severe GVHD is a major cause of NRM and significantly impairs quality of life (QoL) after allo-SCT; therefore, improving GVHD prophylaxis strategies is crucial to reducing mortality and morbidity in these patients. The mechanisms of action of PT-Cy in preventing GVHD after haploidentical T-replete stem cell transplantation remain to be fully defined. However, alloreactive T-cell [31] elimination and regulatory T-cell preservation [32] could partially explain the GVHD prevention after PT-Cy. Different studies have explored PT-Cy-based GVHD prophylaxis regimens in patients receiving allo-SCT from HLAid donors, but mainly in patients with leukemia [9,10,11,12,13,14,15,16,17,18,19] (Table 2). Information on PT-Cy-based GVHD in patients with lymphoma is scant.

This monocentric prospective study focused on the use of PT-Cy-, MPA-, and CSA-based regimens as a GVHD-preventing strategy for patients with lymphoma who underwent an HLAid (both familiar and unrelated) PBSC transplantation with a previous RIC or NMA conditioning. The primary endpoint was achieved, showing a remarkable GRFS rate of 70.4% at three years. Considering the dismal prognosis of a heavily pretreated lymphoma population, the PT-Cy-based prophylaxis regimen after HLAid stem cell transplantation effectively reduced GVHD without losing disease control. With this strategy, few GVHD cases required treatment: only two cases (7.4%) of aGVHD, one grade II and one grade III, and all were responsive to first-line steroids without any further complication. Similarly, only two patients (7.7%) experienced moderate cGVHD and required treatment. These findings translate into a satisfying rate of NRM (7.4%). Despite the very high-risk lymphoma population, the incidence of relapse was low. Among the two patients who relapsed, only one died of progressive disease, and the second is still alive thanks to CAR T-cell therapy rescue. As expected, a longer median time (21 days) to engraftment of neutrophils was observed, probably due to MPA, comparable with a haploidentical transplant, but this did not translate into an increase in infectious events and toxic deaths. It should be noted that only 26% of patients enrolled in our study had undergone letermovir prophylaxis. Therefore, increased use of letermovir [33] over the years will likely further reduce CMV reactivations and infections.

Compared to the study by Broers et al. [34], with all the limits of cross-study comparisons, the main difference in acute and chronic GVHD seems to be using a three-drug prophylaxis regimen in our cohort, superimposable to the haploidentical one used by the Baltimore group [35]. Furthermore, the results of this study are comparable to those recently published in a retrospective series of the European Society for Blood and Marrow Transplantation (EBMT) and the Center for International Blood and Marrow Transplant Research (CIBMTR) [36]. In this retrospective study, matched unrelated donors with PT-Cy were associated with prolonged OS compared with the haploidentical donor, thanks to lower NRM and GVHD incidences. Currently, the patient population with lymphoma has changed, as the majority of patients with lymphoma undergoing allo-SCT are those who have relapsed or are refractory to CAR-T-cell therapy. Therefore, this transplant approach could be applied to this category of patients, given the excellent results achieved in terms of disease relapse and toxicity.

This study has several limitations that must be considered when interpreting the results. Firstly, the relatively small sample size. Second, the short follow-up period limits our ability to assess long-term outcomes and the duration of observed effects. Finally, the absence of a control arm in a single-center study design introduces a potential bias so that more studies would be needed for a more complete evaluation of the efficacy and safety of our approach.

Overall, this study shows that the use of the original Baltimore approach based on three drugs (PT-Cy, CSA, and MPA) could be as effective and safe as the standard approach in the prevention of acute and chronic GVHD also in patients with lymphoma who underwent HLAid PBSC transplantation.

## Figures and Tables

**Table 1:** Patient disease and transplant characteristics.

Characteristics	*n* (%)
Sex:	
Male	15 (56)
Female	12 (44)
Diagnosis:	
HL	9 (33)
NHL	18 (67)
Stage:	
I/II	3 (11)
III/IV	24 (89)
Bulky disease:	
Yes	3 (11)
No	24 (89)
HCT-CI	
Median (IQ range)	2 (1; 3)
Indication for allo-SCT:	
Relapse after autologous	10 (37)
Primary refractory	12 (45)
No mobilize PBSC	2 (7)
Auto–allo program	2 (7)
Frontline therapy	1 (4)
Disease status before HSCT:	
CR	16 (73)
PR	6 (27)
Donor:	
MUD	9 (33)
Sibling	18 (67)
Source:	
BM	1 (4)
PBSC	27 (96)
Conditioning:	
TBF RIC	20 (74)
Baltimora	4 (15)
GITMO-modified	3 (11)

Allo-SCT: allogeneic stem cell transplantation; BM: bone marrow; CR: complete response; GITMO: Gruppo Italiano Trapianto di Midollo Osseo; HL: Hodgkin lymphoma; HSCT: hematopoietic stem cell transplantation; MUD: matched, unrelated donor; NHL: non-Hodgkin lymphoma; PBSC: peripheral blood stem cell; PR: partial response; TBF RIC: thiotepa–busulfan–fludarabine reduced-intensity conditioning.

**Table 2:** Studies with available data of PT-Cy in HLAid-related or unrelated donor transplantation.

Study	Patients (*n*)	Disease	Source	Conditioning	Donor	GVHD Prophylaxis	2–4 aGVHD 3–4 aGVHD	cGVHD	NRM	Relapse
Luznik et al. [13], phase I/II	117	Ly 16% AL 70%	BM	MAC	MRD 67% MUD 33%	PT-Cy alone	42% vs. 46% 12% vs. 8%	9% vs. 11%	2 years 13% vs. 21%	44% 26% (CR)
Kanakry et al. [6], retrospective	209	AL 30%	BM	MAC	MRD 57% MUD 43%	PT-Cy alone	39% vs. 54% 9% vs. 14%	8% vs. 20%	1-year 15%	36% 30% (CR)
Kanakry et al. [12], phase II	92	AL 89% Ly 4%	BM	MAC	MRD 48% MUD 52%	PT-Cy alone	42% vs. 60% 11% vs. 19%	14%	100 days 9% vs. 16%	22%
Mielcarek et al. [14], phase II	43	AL 70% NHL 2%	PBSC	MAC	MRD 28% MUD 72%	PT-Cy + CSA	77% 0%	16%	14% vs. 17%	NA
McCurdy et al. [7], retrospective	321	AL 60% Ly 14%	BM	MAC	MRD 62% MUD 38%	PT-Cy + MPA + FK	18% vs. 23%	7% vs. 15%	28%	NA
Carnevale-Schianca et al. [8], retrospective	35	AL 60% Ly 11%	PBSC	MAC RIC	MUD 57% 9/10 23% 8/10 20%	PT-Cy + MPA + FK	17% vs. 12%	7%	3%	46% 25% (CR)
Rashidi et al. [9],	21	AML	PBSC	MAC RIC	MRD MUD	PT-Cy + MPA + FK	19%	5%	16%	26%
retrospective										
Ruggeri et al. [15], retrospective	423	AL	PBSC BM	MAC RIC	MRD MUD	PT-Cy alone PT-Cy + MPA/CSA/MTX PT-Cy + CSA + MPA/ MTX	27.9%	18% 20% 8.5%	19% 20% 14%	32% 36% 28%
Luznik et al. [16], phase III	346	AL MDS CMML	PBSC BM	MAC RIC	MRD MUD	CD34+ selection PT-Cy MTX + FK	16.3% vs. 37.6% vs. 29.8% 2.9% vs. 10.1% vs. 3.5%	8.9% 27% 33.7%	21.5% 15.7% 7.9%	21.4% 13.9% 25.6%

aGVHD: acute graft-versus-host disease; cGVHD: chronic graft-versus-host disease; NRM: non-relapse mortality; Ly: lymphoma, AL: acute leukemia; AML: acute myeloid leukemia; MDS: myelodysplastic syndrome; CMML: chronic myelomonocytic leukemia; BM: bone marrow; CR: complete response; PBSC: peripheral blood stem cell; MAC: myeloablative conditioning regimen; RIC: reduced-intensity conditioning; MUD: matched, unrelated donor; MRD: matched, related donor; PT-Cy: post-transplant cyclophosphamide; CSA: cyclosporine; MPA: mycophenolic acid; FK: tacrolimus; MTX: methotrexate; NA: not available.

## Data Availability

The data that supports the findings of this study are available on request from the corresponding author, [D.T.]. The data are not publicly available due to their containing information that could compromise the privacy of research participants.
